# Beyond the Diagnosis: A Comprehensive Case Report on Duchenne Muscular Dystrophy

**DOI:** 10.1155/crcc/9969987

**Published:** 2026-09-07

**Authors:** Mahmoud Abdel Hameed Shahin, Atheer Ali Asiri, Areej Salah Alotaibi, Huda Majrashi, Saeedah Khubrani, Shorouq Hashel AlQarni, Shomokh Saad Alqurashi, Manal Essa Samti, Zahra Hashel AlQarni

**Affiliations:** ^1^ Nursing Department, Prince Sultan Military College of Health Sciences, Dhahran, Eastern Region, Saudi Arabia; ^2^ Preventive Medicine Program Department, Armed Forces Hospital Southern Region, Abha City, Saudi Arabia, afhsr.med.sa

**Keywords:** calf hypertrophy, Duchenne muscular dystrophy, muscle wasting, proximal muscle weakness, X-linked muscular dystrophy

## Abstract

**Background:**

Duchenne muscular dystrophy (DMD) is an X‐linked recessive genetic disorder characterized by the absence of dystrophin, leading to progressive muscle degeneration and generalized weakness. It primarily affects males in early childhood and progresses to loss of ambulation and cardiac and respiratory complications.

**Case Report:**

This case report describes a 26‐year‐old male with terminal‐stage DMD who was admitted to the surgical intensive care unit (SICU) in a critically ill state with multiple‐organ failure. He was ventilator‐dependent via tracheostomy, hypotensive, anemic, and required cardiovascular support. He also had multiple pressure ulcers, including a Stage IV ulcer with associated edema, acute renal failure managed with continuous renal replacement therapy (CRRT), and sepsis due to mixed bacterial and fungal infections. Broad‐spectrum antimicrobial therapy and intensive wound care were provided. This case highlights that advanced‐stage DMD is a severe multisystem condition requiring intensive, specialized, and multidisciplinary care.

**Conclusion:**

This case underscores the critical importance of a multidisciplinary approach in managing complex patient conditions, emphasizing the prevention of secondary complications such as pressure ulcers and pulmonary infections. Adherence to evidence‐based guidelines, coupled with psychosocial and ethical considerations related to quality of life, remains essential. Establishing standardized care protocols, enhancing healthcare team training on the specific needs of these patients, and promoting home‐based and palliative care services are strongly recommended to ensure comprehensive, coordinated, and patient‐centered management.

## 1. Introduction

### 1.1. Overview of Duchenne Muscular Dystrophy (DMD)

**Figure 1:**
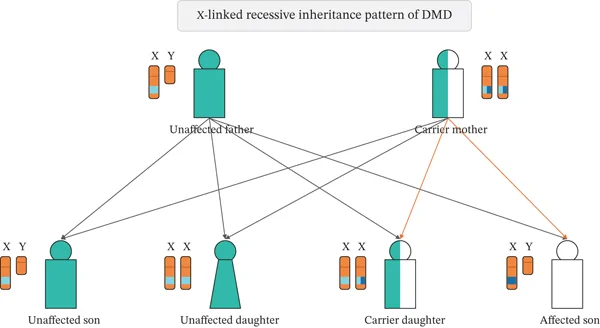
X‐linked recessive inheritance pattern of DMD.

DMD is a severe, X‐linked recessive neuromuscular disorder primarily affecting boys, caused by mutations in the dystrophin gene on chromosome Xp21, leading to the absence or severe deficiency of the dystrophin protein. This results in progressive skeletal muscle weakness, degeneration, and eventual respiratory and cardiac complications, with symptoms typically emerging in early childhood and a life expectancy into the late 20s to early 30s with modern care [1]. Duchenne can be inherited from a parent or result from a random, spontaneous genetic change that may occur during any pregnancy [2]. In fact, about one in three cases occurs in families with no prior history of Duchenne (Figure 1).

### 1.2. Case History and Clinical Context

The patient of this report has had DMD since childhood, with progressive respiratory failure leading to mechanical ventilation, acute renal failure requiring continuous renal replacement therapy (CRRT), systemic sepsis with multimicrobial infection, and multiple advanced pressure ulcers. He was admitted to the surgical intensive care unit (ICU) of a military hospital in the eastern region of Saudi Arabia on October 23, 2025, with the primary complaint being that the patient, a 26‐year‐old male with advanced DMD, was hospitalized, completely dependent on a ventilator, and suffering from multiple organ failure. His previous medical history includes chronic complications such as respiratory failure (mechanical ventilator dependent since September 29, 2025), dilated cardiomyopathy, mild to moderate cognitive impairment, recurrent pneumonia, and a previous tracheostomy.

On physical examination, the patient was very thin, partially unconscious (Glasgow Coma Scale score 10/15), with generalized edema and severe muscle wasting. Multiple Grade IV pressure ulcers were found on the buttocks and thighs, along with inflammation and swelling of the skin. Vital signs showed hypotension (95/66 mmHg) and a heart rate of 78 bpm. Hematuria and oliguria were also noted. Laboratory tests revealed severe anemia (hemoglobin 7.98 g/dL), markedly elevated creatine kinase, positive cultures for *Pseudomonas aeruginosa* and *Burkholderia* in the airway, and fungal growth in the urine.

Echocardiography confirmed cardiomyopathy, whereas previous genetic testing and muscle biopsy documented mutations in the dystrophin gene and dystrophin protein deficiency. Continuous monitoring included blood gasses, vital signs, and renal function. The diagnosis was end‐stage DMD, accompanied by acute respiratory failure, sepsis, acute renal failure (requiring ongoing renal replacement therapy), severe anemia, advanced pressure ulcers, cardiomyopathy, generalized edema, skin infection, and malnutrition. Admission is aimed at managing the acute, multiple organ complications through intensive, multidisciplinary care.

#### 1.2.1. Genetic and Familial Assessment

The diagnosis was clinically supported by prior genetic testing and muscle biopsy findings, which confirmed a dystrophin gene mutation and corresponding protein deficiency. However, the specific variant nomenclature, transcript or isoform reference, and the complete genetic laboratory report were not available in the reviewed clinical records. The patient had a documented history of two older brothers affected by DMD who died from disease‐related complications. No additional family history of affected maternal uncles or other male relatives was identified in the available documentation. This gap is clinically significant given the well‐established hereditary nature of DMD. Identification of a pathogenic DMD variant typically warrants carrier testing and genetic counseling for the mother and at‐risk female relatives to facilitate informed reproductive decision‐making, cascade testing, and early detection in other potentially affected family members.

## 2. Brief Review of Recent Research

### 2.1. Progression of DMD and Multisystem Involvement

The study by Suslov et al. [3] examined the development of functional status in children with DMD, focusing on the impact of physical therapy on the disease′s progression. The study demonstrated that the disease is characterized by a progressive, continuous decline in muscle strength and function, and that although physical therapy may slow this decline in the early stages, it does not alter the disease′s inevitable progressive course, thus confirming the ongoing degenerative nature of DMD.

The randomized controlled trial by Hernández‐Sánchez et al. [4] aimed to evaluate the role of family involvement and home‐based physical therapy in patients with DMD. The results showed a relative improvement in motor performance among patients who received family‐supported home‐based therapy compared with those who received conventional therapy alone. However, this improvement did not prevent long‐term functional decline, supporting the notion that supportive interventions improve function temporarily without affecting the overall progression of the disease [4].

In a multicenter study, Muntoni et al. [5] aimed to identify clinically significant functional changes in patients with DMD to track disease progression more accurately. Their findings confirmed that motor function changes gradually and can serve as a reliable indicator of disease progression, reinforcing the understanding of DMD as a long‐term, progressive disease that ultimately leads to complete motor loss.

Regarding the pathogenesis, a study by Cardone et al. provided a comprehensive analysis of the histological pathways of changes in Duchenne‐affected muscles, demonstrating that muscle regeneration failure results from the senescence of muscle stem cells. These findings helped explain the persistent and irreversible decline in muscle mass, directly linking the cellular basis to the clinical progression of the disease [6]. In a narrative review, Abid et al. [7] discussed the roles of both physical therapy and gene therapy in the management of muscle atrophies, with a focus on DMD. The review indicated that current supportive therapies improve symptoms and delay complications, but do not halt disease progression. Gene therapies, meanwhile, are still in the research phase and have not yet demonstrated long‐term clinical efficacy [7].

Patterson et al. [8] also provided a comprehensive review of current and emerging therapies, including exon skipping and gene therapy. They explained that these strategies represent significant advances in DMD care, but have not yet succeeded in preventing progression to advanced stages or reducing organ involvement, underscoring the continued multisystem nature of the disease. Finally, Budzynska et al. [9] discussed the potential of chimeric cell therapy as an innovative approach to restoring dystrophin expression and stimulating muscle regeneration. Despite promising experimental results, the study concluded that these strategies are still in the research phase and have not yet demonstrated clinical efficacy in halting multisystem deterioration in DMD [9].

### 2.2. Critical Care Management and Complications in Advanced DMD

In advanced DMD patients, critical care management involves addressing severe respiratory, infectious, and renal complications. Mechanical ventilation plays a central role in managing respiratory failure, with modern strategies such as APRV and Bilevel ventilation tailored to each patient′s respiratory mechanics to optimize treatment efficacy and minimize complications, highlighting the importance of individualized ventilatory support in critically ill DMD patients [10]. Additionally, the application of artificial intelligence in ventilator management has been shown to improve ventilator settings, reduce errors, and enhance adherence to clinical guidelines, thereby strengthening patient outcomes in ICUs for patients requiring prolonged mechanical ventilation [11].

Sepsis remains a significant threat in advanced DMD, where early recognition and implementation of evidence‐based intensive treatment protocols are critical to reduce mortality and complications [12]. The Surviving Sepsis Campaign 2021 emphasizes comprehensive assessment and timely intervention, along with long‐term monitoring of sepsis‐related complications, reinforcing the need for guideline‐based management in these vulnerable patients [13].

Acute kidney injury (AKI) is another frequent complication in critically ill patients, with high incidence and mortality in intensive care settings. Studies show that early monitoring and renal support are vital for DMD patients at risk [14]. Moreover, research on burn patients demonstrates that prompt intervention can mitigate AKI‐related complications, principles that are directly applicable to multiple organ‐compromised DMD patients [15]. Finally, machine learning approaches have shown potential for predicting early AKI onset in septic patients, enabling timely intervention and improved intensive care outcomes, which are crucial for the management of advanced DMD with multiple organ involvement [16].

### 2.3. Multidisciplinary and Palliative Care in End‐Stage DMD

In the management of end‐stage DMD, a multidisciplinary approach is essential to optimize patient outcomes and quality of life. Recent literature emphasizes that coordinated care involving neurology, pulmonology, cardiology, nephrology, physiotherapy, nursing, and social services reduces complications and enhances overall patient management [17, 18]. Multidisciplinary strategies facilitate early intervention, comprehensive monitoring, and tailored treatment plans, which are particularly crucial in complex cases with multiple organ involvement, as seen in advanced DMD.

Palliative care also plays a central role in these patients, focusing on symptom relief, psychosocial support, and decision‐making concordance between patients and families. Studies demonstrate that involving caregivers in care decisions improves adherence, patient satisfaction, and emotional well‐being, even when disease progression cannot be halted [19, 20]. Hospice and community‐based palliative services provide social support and maintain patient dignity, further contributing to the quality of life [21]. Moreover, research on quality‐of‐life scales highlights the importance of assessing the physical, psychological, and social dimensions to guide interventions that enhance patient‐centered outcomes [22–24]. Collectively, these studies underscore that multidisciplinary care, palliative support, and quality‐of‐life monitoring are interdependent pillars in the holistic management of end‐stage DMD.

## 3. Case Description

Table 1 provides a structured overview of a 26‐year‐old male patient with end‐stage DMD, highlighting demographic data, chief complaints, current and prior medical history, medications, clinical findings, laboratory and radiologic investigations, differential diagnoses, and the definitive diagnosis, including major complications. The format provides a quick, clear understanding of the patient′s complex condition and facilitates clinical decision‐making by organizing symptoms, test results, and complications.

**Table 1:** Demographic and medical data.

Item	Summary
Age and sex	26‐year‐old male
Principal complaint	Advanced muscle weakness, acute respiratory failure, and multiple organ involvement (cardiac, renal, and skin).
Current medical history	Late‐stage DMD, bedridden, fully dependent on mechanical ventilation via tracheostomy; complications include: respiratory (respiratory failure, pulmonary sepsis), cardiovascular (cardiomyopathy, blood pressure support), renal (acute kidney injury, CRRT), skin (multiple pressure ulcers, dermatitis), multisite infections, severe muscle wasting.
Previous medical history	Confirmed DMD based on clinical history, previous genetic testing, and muscle biopsy showing dystrophin deficiency. Exact genetic variant nomenclature and transcript/isoform reference were not available in the reviewed records; no documented family history of affected male relatives was found.
Medications	Antibiotics (ceftolozane), blood pressure support (Levophed), anticoagulants (heparin for DVT prophylaxis), analgesics, nutritional and electrolyte support as needed. Available records did not document current or prior corticosteroid therapy, nor did they document the use of an HDAC inhibitor, such as givinostat.
Clinical examination	Bedridden, partially conscious (GCS 10/15), pale, edematous, severe muscle wasting, tracheostomy (8 mm) connected to mechanical ventilation, moderate airway secretions, SpO_2_ 97%, HR 78 bpm, BP 95/66 mmHg, multiple pressure ulcers, dermatitis, complete motor weakness.
Laboratory tests	Low hemoglobin (7.98 g/dL), positive cultures: urine (*Candida*), trachea (*Burkholderia* and *Pseudomonas*), wound (*Pseudomonas*), markedly elevated CK, ABG: hypoxemia (PaO_2_ 47–49 mmHg).
Radiological investigations	Echocardiography (dilated cardiomyopathy), chest x‐ray (lung and respiratory infection assessment), possible spine imaging or other studies, depending on complications.
Differential diagnosis	Acute respiratory failure (neuromuscular weakness vs. severe pulmonary infection), sepsis (multisource), multiple organ failure, other muscular dystrophies or neuromuscular disorders.
Definitive diagnosis	End‐stage Duchenne muscular dystrophy with acute respiratory failure requiring mechanical ventilation, suspected/confirmed sepsis, acute kidney injury (on CRRT), cardiomyopathy, multiple advanced pressure ulcers, severe malnutrition, and cachexia.
Genetic and familial assessment	Previous testing supported dystrophin gene involvement, but the original report with variant nomenclature was unavailable. Family history was partly documented in the reviewed records; genetic counseling and carrier testing for at‐risk female relatives are recommended when a pathogenic DMD variant is confirmed.

## 4. Statistical Analysis

No inferential statistical analysis was performed because this manuscript is a single‐patient case report. Quantitative clinical findings are presented descriptively using the original measurement units where available, including vital signs, Glasgow Coma Scale score, hemoglobin level, arterial oxygen tension, oxygen saturation, creatine kinase status, and culture results. No *p* values, confidence intervals, means, medians, or measures of dispersion were calculated because no comparative group or repeated analytic dataset was analyzed.

## 5. Clinical Manifestations

### 5.1. Symptoms

DMD patients usually present in early childhood with developing motor problems. Common symptoms include delayed motor milestones, frequent stumbles, difficulty getting up from the floor (Gowers′ maneuver), and toe walking due to proximal muscle weakness. As the disease progresses, respiratory symptoms such as dyspnea and impaired exercise tolerance appear, finally leading to full loss of voluntary movement and ventilator dependency in the late stages. Additional symptoms in advanced disease may include widespread weariness, muscular and joint discomfort, and, in later stages, dysphagia and speech difficulties due to bulbar and respiratory muscle involvement.

### 5.2. Clinical Signs

Clinical examination reveals distinctive features, including pseudohypertrophy of the calf muscles, generalized muscular atrophy of both upper and lower limbs, joint contractures, and spinal abnormalities such as scoliosis. Advanced phases are distinguished by peripheral edema, hypoxemia‐induced cyanosis, and the utilization of supplementary respiratory muscles. Immobilized individuals typically develop many pressure sores and skin disintegration. A positive Gowers′ sign is a key finding in the early stages, indicating substantial proximal muscle weakness.

### 5.3. Clinical Progression

DMD follows a predictable, relentlessly progressive course. Symptoms often appear between the ages of 2 and 5 years, with a gradual loss of ambulation happening around the ages of 10 and 12. Progressive respiratory muscle weakness leads to chronic respiratory insufficiency and ultimately to reliance on mechanical ventilation. Cardiac involvement progresses from subclinical cardiomyopathy to outright heart failure. Most patients die of respiratory or cardiac failure in early adulthood in the absence of effective disease‐modifying medication; however, breakthroughs in supportive care have modestly extended survival [25].

### 5.4. Unusual Manifestations

In addition to the usual neuromuscular characteristics, patients may have mild to moderate cognitive or learning impairments. AKI and severe multisystem infections are common atypical symptoms in advanced and complicated cases, generally as a result of protracted critical illness, sepsis, or multiple organ failure. Opportunistic infections with organisms such as *Burkholderia*, *Pseudomonas*, and *Candida* indicate immunological compromise and protracted invasive medical care [26].

### 5.5. Management

DMD management is primarily supportive and requires a multidisciplinary approach. Respiratory care includes noninvasive or invasive mechanical ventilation, regular arterial blood gas monitoring, and prompt treatment of respiratory illnesses. Immobilized individuals require proper skin care and pressure ulcer prevention. Comprehensive management also requires coordinated input from neurology, pulmonology, cardiology, nephrology, rehabilitation, nutrition, and mental health services, with a greater emphasis on palliative care in end‐stage disease [27].

### 5.6. Treatment Plan

Antibiotics are used to treat active infections, vasopressors to treat hemodynamic instability, anticoagulants to prevent thrombosis, and analgesics to relieve pain. Corticosteroids (e.g., prednisone or deflazacort) are often used to halt disease progression in the early stages. Emerging treatments, such as exon‐skipping and gene‐based therapy, offer potential benefits but do not yet prevent progression to advanced multisystem involvement. Standard heart failure drugs are used to treat cardiomyopathy. In the present case, the available records did not document prior or current corticosteroid treatment. Because long‐term glucocorticoid therapy is commonly used in DMD to delay loss of ambulation and some secondary complications, absence or delayed use may plausibly contribute to earlier functional decline; however, causality cannot be inferred from this single case without full longitudinal treatment records. HDAC inhibitors such as givinostat are emerging disease‐modifying therapies for DMD. Still, they are mainly relevant earlier in the disease course, and their ability to prevent terminal multisystem failure in an end‐stage, ventilator‐dependent adult remains uncertain.

### 5.7. Medical and Surgical Interventions

Advanced DMD frequently requires invasive procedures, such as tracheostomy for long‐term ventilatory support and CRRT for acute kidney failure. Surgical and procedural interventions may also include debridement of necrotic pressure ulcers, urine catheterization, nasogastric or gastrostomy tube implantation for nutritional support, and in some situations, orthopedic procedures including spine stabilization.

### 5.8. Outcome and Follow‐Up

DMD′s overall prognosis remains dismal, especially in end‐stage illness marked by multiple organ failure and recurrent sepsis. Because the disease is permanent and progressing, management objectives shift to increasing survival, reducing complications, and improving quality of life.

## 6. Discussion

This clinical case clearly reflects the typical progressive course of DMD, as described in recent literature. The disease gradually progresses from progressive muscle weakness in childhood to a complex systemic condition culminating in multiple organ failure in early adulthood. The presentation aligns with the findings of Suslov et al. [3], who confirmed that supportive interventions, including physical therapy and respiratory support, can delay the onset of some complications but do not halt terminal functional decline, prevent muscle mass loss, or protect vital organs from damage. Furthermore, the complete reliance on mechanical ventilation, the presence of dilated cardiomyopathy, and chronic respiratory failure accurately reflect the terminal stage of the disease as reported in long‐term clinical studies.

The rapid deterioration and progression to multiple organ failure can be explained by the intrinsic pathogenesis of DMD, namely the complete absence of dystrophin protein, which leads to muscle cell membrane fragility and failure of muscle regeneration [28]. This structural defect causes progressive deterioration of skeletal, respiratory, and cardiac muscles, which explains the development of chronic respiratory failure and the patient′s dependence on long‐term mechanical ventilation. In addition, dilated cardiomyopathy contributed to hemodynamic instability, increasing the risk of organ ischemia. Severe malnutrition, muscle wasting, and prolonged immobility also contributed to a weakened immune system and increased susceptibility to severe and recurrent infections.

Systemic involvement in DMD can be understood through two complementary mechanisms. First, chronic dystrophin deficiency in skeletal muscles leads to progressive weakness, immobility, respiratory muscle failure, malnutrition, increased risk of pressure injuries, and increased vulnerability to infection. Second, dystrophin is also expressed in nonskeletal tissues, including cardiac muscle and the central nervous system; therefore, cardiomyopathy, cognitive involvement, and dysregulated multisystem responses may reflect both long‐standing skeletal muscle disease and tissue‐specific dystrophin deficiency [1, 25]. In this patient, dilated cardiomyopathy, chronic respiratory failure, cognitive impairment, renal failure during sepsis, and severe pressure ulcers illustrate how DMD evolves from a primary neuromuscular disorder into a systemic critical illness.

Advanced pressure ulcers in this case are a predictable consequence of the interaction of several factors, including complete paralysis, impaired peripheral blood flow, protein malnutrition, and prolonged immobility without effective positioning. The infection was multifocal, involving the respiratory, wound, and urinary tracts, with the isolation of opportunistic bacteria and fungi. This is consistent with the literature on the increased risk of complex infections in end‐stage DMD patients due to prolonged critical care and repeated medical interventions [29]. In contrast, acute kidney failure is interpreted as an intervening consequence of sepsis, hypotension, use of nephrotoxicating drugs, and possibly myolysis, necessitating CRRT in accordance with best practices for multiple organ failure [30].

The treatment plan followed was largely consistent with current recommendations for managing patients in ICUs, including long‐term mechanical ventilation, broad‐spectrum antibiotic therapy based on transplant results, vasopressor support, and CRRT [31]. However, causative treatment options were minimal due to the advanced stage of the disease, reflecting the irreversible nature of DMD at this stage. This case suggests that earlier palliative care could have improved quality of life, reduced the treatment burden, and supported the family in making treatment decisions that balanced life prolongation with meaning.

Regarding disease‐modifying therapy, the reviewed records did not show that the patient was currently or previously treated with corticosteroids. This limits interpretation because steroid exposure, dose, duration, adherence, and timing may influence long‐term ambulation, respiratory function, scoliosis, and cardiac outcomes. Similarly, givinostat (Duvyzat), an HDAC inhibitor approved by the U.S. Food and Drug Administration for patients aged 6 years and older with DMD, represents an important newer nonsteroidal option; however, evidence mainly supports slowing disease progression rather than reversing advanced organ failure. In a terminal‐stage adult who is ventilator‐dependent and complicated by sepsis, AKI, pressure ulcers, and cardiomyopathy, the expected benefit of starting HDAC inhibition would likely be limited and would require specialist assessment of safety, platelet count, gastrointestinal tolerance, infection burden, and overall goals of care [32, 33].

It is also important to consider whether the observed outcome could have been prevented by restoring dystrophin expression only in skeletal muscles or by preventing cardiac and/or brain involvement. Skeletal muscle‐targeted treatment might delay weakness, immobility, respiratory decline, and pressure ulcer risk, but it would not fully address cardiac dystrophin deficiency or neurocognitive involvement. Conversely, earlier cardiac surveillance and cardioprotective treatment may reduce cardiomyopathy‐related deterioration but would not prevent skeletal muscle wasting [34]. Therefore, the systemic nature of advanced DMD supports the need for early whole‐patient monitoring and combined neuromuscular, respiratory, cardiac, nutritional, skin, psychosocial, and palliative care rather than focusing on a single organ system.

The main challenges in managing this condition stem from the inevitable progressive nature of the disease, the multitude of overlapping systemic complications, the lengthy ICU stay, and the ethical complexities associated with the balancing act of intensive supportive care and the resulting low quality of life. The lack of clear, standardized protocols for managing end‐stage DMD further complicates clinical decision‐making and underscores the need for more specific guidelines. This case highlights the critical importance of early intervention and long‐term follow‐up within a multidisciplinary team to delay complications, reduce their severity, and improve quality of life for DMD patients.

It also underscores the necessity of implementing proactive preventive strategies, particularly regarding pressure ulcer prevention, close monitoring for infections, and early planning of palliative care as an integral part of the overall treatment plan. Based on the findings of this case study, the research recommends strengthening investigations into gene and cell therapies that target the root cause of the disease, as well as developing integrated care models that include home‐based and palliative care. It also recommends establishing clear, standardized protocols for managing patients with advanced DMD to ensure structured, compassionate, and evidence‐based care.

## 7. Conclusion

This case of end‐stage DMD underscores the profound and irreversible multisystem deterioration that defines the terminal phase of this devastating neuromuscular disorder. Despite the application of advanced intensive care measures, the patient developed progressive respiratory failure requiring long‐term mechanical ventilation, severe sepsis of multifactorial origin, AKI necessitating CRRT, and extensive pressure ulcers, collectively illustrating the cumulative burden of dystrophin deficiency and prolonged critical illness. These findings reinforce the well‐established understanding that, in advanced DMD, supportive therapies can mitigate complications but cannot alter the inevitable trajectory toward multiple organ failure.

The key message highlighted by this case is that the management of advanced DMD must extend beyond organ‐specific interventions to embrace early, integrated, and multidisciplinary care pathways that prioritize holistic patient needs. Timely incorporation of palliative care is essential—not as an alternative to active treatment, but as a complementary approach that supports symptom control, psychosocial well‐being, ethical decision‐making, and alignment of care goals with patient and family values. In the absence of curative therapies, preserving dignity, minimizing suffering, and maintaining quality of life should remain central objectives of care.

Several practical implications emerge from this case. First, there is a critical need to develop and implement standardized, evidence‐based, multidisciplinary care protocols for patients with DMD across all disease stages, with particular emphasis on anticipatory management of respiratory, cardiac, renal, and skin‐related complications. Second, enhanced education and training of healthcare professionals are necessary to improve recognition, prevention, and early management of the complex complications associated with advanced neuromuscular disorders. Third, the early integration of palliative care principles should be normalized across the care continuum for DMD, facilitating shared decision‐making, supporting families during prolonged illness trajectories, and reducing the emotional and ethical burden of end‐of‐life care.

In addition, strengthening home‐based and community‐centered care models represents a pivotal strategy to reduce recurrent hospitalizations, optimize continuity of care, and promote patient‐centered outcomes. Such models may improve symptom management, reduce strain on the healthcare system, and better address the long‐term needs of patients living with advanced DMD and their caregivers. Looking to the future, this case highlights the urgent need for continued and intensified research into disease‐modifying strategies, including gene editing, exon‐skipping technologies, and emerging molecular and cellular therapies aimed at restoring dystrophin expression or modifying disease progression. Although these approaches hold promise, their clinical impact remains limited at present, underscoring the parallel necessity for health systems to evolve toward more coordinated, compassionate, and sustainable care pathways. Ultimately, improving outcomes for individuals with DMD will require not only scientific innovation but also a commitment to integrated, humane, and patient‐centered care throughout the entire disease course.

## Author Contributions

M.A.H.S. (corresponding author) conceived the idea of the study, supervised the research process, and drafted the initial version of the manuscript. A.A.A., A.S.A., H.M., S.K., S.H.A., S.S.A., M.E.S., and Z.H.A. contributed equally to various aspects of the work, including data collection, data interpretation, patient examination and record review, manuscript revision, final draft preparation, and proofreading.

## Funding

No funding was received for this manuscript.

## Disclosure

All authors read and approved the final version of the manuscript.

## Ethics Statement

Ethics approval was not required; however, a written informed consent was obtained from the patient, who was admitted to King Fahd Military Medical Complex‐Dhahran, Saudi Arabia, before data collection for both the case study and for publication of the details of their medical case and any accompanying images of this case report (on October 26th, 2025). The study was conducted in accordance with the ethical principles of the Declaration of Helsinki and all applicable institutional and national research ethics guidelines.

Patient anonymity and confidentiality were strictly maintained. No personally identifiable information has been reported, and all data have been deidentified to protect the patient′s privacy. All patient data are stored securely on the password‐protected computer of the principal investigator and will not be disclosed to any unauthorized parties.

## Conflicts of Interest

The authors declare no conflicts of interest.

## Data Availability

The data that support the findings of this study are available from the corresponding author upon reasonable request.

## References

[1] Venugopal V. and Pavlakis S. , Duchenne Muscular Dystrophy, StatPearls Treasure Island, 2023, StatPearls Publishing LLC., http://www.ncbi.nlm.nih.gov/pubmed/29493971. 29493971

[2] Parent Project Muscular Dystrophy , What is Duchenne, 2026, Firefly Partners, http://www.parentprojectmd.org/about-duchenne/what-is-duchenne/.

[3] Suslov V. M. , Lieberman L. N. , Suslova G. A. , Bure N. P. , Adulas E. I. , and Rudenko D. I. , Physical Therapy in Patients With Duchenne Muscular Dystrophy: Dynamics of the Course of the Disease, Pediatrician. (2022) 13, no. 3, 37–46, 10.17816/PED13337-46.

[4] Hernández-Sánchez A. , Parra-Sánchez L. , Montolio M. , Rueda-Ruzafa L. , Ortiz-Comino L. , and Sánchez-Joya M. D. M. , Family Involvement and at-Home Physical Therapy on Duchenne Muscular Dystrophy: A Randomized Controlled Trial, Pediatric Neurology. (2024) 152, 34–40, 10.1016/j.pediatrneurol.2023.12.015, 38184986. 38184986

[5] Muntoni F. , Signorovitch J. , Sajeev G. , Done N. , Yao Z. , Goemans N. , McDonald C. , Mercuri E. , Niks E. H. , Wong B. , and Vandenborne K. , Meaningful Changes in Motor Function in Duchenne Muscular Dystrophy (DMD): A Multi-Center Study, PLoS One. (2024) 19, no. 7, e0304984, 10.1371/journal.pone.0304984. 38985784 PMC11236155

[6] Cardone N. , Taglietti V. , Baratto S. , Kefi K. , Periou B. , Gitiaux C. , Barnerias C. , Lafuste P. , Pharm F. L. , Pharm J. N. , and Panicucci C. , Myopathologic Trajectory in Duchenne Muscular Dystrophy (DMD) Reveals Lack of Regeneration due to Senescence in Satellite Cells, Acta Neuropathologica Communications. (2023) 11, no. 1, 10.1186/s40478-023-01657-z. PMC10585739 37858263

[7] Abid H. , Shah S. , Ahmed A. , Habib N. , Bibi M. , and Ibrahim M. , Role of Physical Therapy Intervention and Gene Therapy on Muscular Dystrophies, Current Status and Future Perspectives: A Narrative Review, Pakistan Journal of Health Sciences. (2024) 5, 9–14, 10.54393/pjhs.v5i01.1253.

[8] Patterson G. , Conner H. , Groneman M. , Blavo C. , and Parmar M. S. , Duchenne Muscular Dystrophy: Current Treatment and Emerging Exon Skipping and Gene Therapy Approach, European Journal of Pharmacology. (2023) 947, 175675, 10.1016/j.ejphar.2023.175675. 36963652

[9] Budzynska K. , Siemionow M. , Stawarz K. , Chambily L. , and Siemionow K. , Chimeric Cell Therapies as a Novel Approach for Duchenne Muscular Dystrophy (DMD) and Muscle Regeneration, Biomolecules. (2024) 14, no. 5, 10.3390/biom14050575. PMC11117592 38785982

[10] Hickey S. M. , Sankari A. , and Giwa A. O. , Mechanical Ventilation, StatPearls Treasure Island, 2025, StatPearls Publishing LLC, http://www.ncbi.nlm.nih.gov/pubmed/30969564. 30969564

[11] Gallifant J. , Zhang J. , Del Pilar Arias Lopez M. , Zhu T. , Camporota L. , and Celi L. A. , Artificial Intelligence for Mechanical Ventilation: Systematic Review of Design, Reporting Standards, and Bias, British Journal of Anaesthesia. (2022) 128, no. 2, 343–351, 10.1016/j.bja.2021.09.025. 34772497 PMC8792831

[12] Srzić I. , Nesek Adam V. , and Tunjić P. D. , Sepsis Definition: What′s New in the Treatment Guidelines, Acta Clinica Croatica. (2022) 61, no. supplement 1, 67–72, 10.20471/acc.2022.61.s1.11. PMC9536156 36304809

[13] Oczkowski S. , Alshamsi F. , Belley-Cote E. , Centofanti J. E. , Hylander Møller M. , Nunnaly M. E. , and Alhazzani W. , Surviving Sepsis Campaign Guidelines 2021: Highlights for the Practicing clinician, Polish Archives of Internal Medicine. (2022) 132, no. 7-8, 10.20452/pamw.16290, 35791800. 35791800

[14] Nadim M. K. and Garcia-Tsao G. , Acute Kidney Injury in Patients With Cirrhosis, New England Journal of Medicine. (2023) 388, no. 8, 733–745, 10.1056/NEJMra2215289. 36812435

[15] Legrand M. , Clark A. T. , Neyra J. A. , and Ostermann M. , Acute Kidney Injury in Patients With Burns, Nature Reviews Nephrology. (2024) 20, no. 3, 188–200, 10.1038/s41581-023-00769-y. 37758939 PMC13340345

[16] Yue S. , Li S. , Huang X. , Liu J. , Hou X. , Zhao Y. , Niu D. , Wang Y. , Tan W. , and Wu J. , Machine Learning for the Prediction of Acute Kidney Injury in Patients With Sepsis, Journal of Translational Medicine. (2022) 20, no. 1, 10.1186/s12967-022-03364-0, 35562803. PMC9101823 35562803

[17] Alsubaie S. S. , Bukhamseen Z. F. A. , Alyami F. S. A. , Alotaibi M. F. , Alkahtani F. A. , Alkhamsan M. S. , Alaseel A. J. , Albahri M. A. S. , Bahrani J. I. A. A. , and Khamees Z. H. A. , Multidisciplinary Approaches in General Medical Practice: Enhancing Collaboration for Better Patient Care, Journal of Ecohumanism. (2024) 3, no. 7, 2659–2669, 10.62754/joe.v3i7.4665.

[18] Korylchuk N. , Pelykh V. , Nemyrovych Y. , Didyk N. , and Martsyniak S. , Challenges and Benefits of a Multidisciplinary Approach to Treatment in Clinical Medicine, Journal of Pioneering Medical Sciences. (2024) 13, no. 3, 1–9, 10.61091/jpms202413301.

[19] Mulcahy Symmons S. , Ryan K. , Aoun S. M. , Selman L. E. , Davies A. N. , Cornally N. , Lombard J. , McQuilllan R. , Guerin S. , O′Leary N. , Connolly M. , Rabbitte M. , Mockler D. , and Foley G. , Decision-Making in Palliative Care: Patient and Family Caregiver Concordance and Discordance-Systematic Review and Narrative Synthesis, BMJ Supportive & Palliative Care. (2023) 13, no. 4, 374–385, 10.1136/bmjspcare-2022-003525, 35318213. PMC10804031 35318213

[20] Valero-Cantero I. , Casals C. , Carrión-Velasco Y. , Barón-López F. J. , Martínez-Valero F. J. , and Vázquez-Sánchez M. , The Influence of Symptom Severity of Palliative Care Patients on Their Family Caregivers, BMC Palliative Care. (2022) 21, no. 1, 10.1186/s12904-022-00918-3. PMC8886938 35227246

[21] Bradley N. M. , Dowrick C. F. , and Lloyd-Williams M. , A Survey of Hospice Day Services in the United Kingdom & Republic of Ireland: How Did Hospices Offer Social Support to Palliative Care Patients, Pre-Pandemic?, BMC Palliative Care. (2022) 21, no. 1, 10.1186/s12904-022-01061-9. PMC9532229 36195870

[22] Karacan Y. , Akkus Y. , Bayram R. , Budak S. , and Ünlü A. A. , Do Spiritual Well-Being and Pain Intensity Predict Physical or Mental Components of Health-Related Quality-of-Life Scale in Patients With Multiple Myeloma?, Pain Management Nursing. (2024) 25, no. 5, e367–e374, 10.1016/j.pmn.2024.05.006. 38834417

[23] Mason D. , Rodgers J. , Garland D. , Wilson C. , Parr J. R. , and McConachie H. , Measuring Quality of Life in Autistic Adults: The Reliability and Validity of the Brief Version of the World Health Organization Quality of Life scale, AMRC Open Research. (2022) 4.

[24] Yang K. , Wang X. , Hu S. , Li Y. , Lei T. , Li Y. , and Cui Y. , The Psychometric Properties of Chinese Version of the Gilles de la Tourette Syndrome-Quality of Life Scale (GTS-QOL) for Children and Adolescents, BMC Psychiatry. (2024) 24, no. 1, 10.1186/s12888-024-06149-5, 39443913. PMC11515785 39443913

[25] Bez Batti Angulski A. , Hosny N. , Cohen H. , Martin A. A. , Hahn D. , Bauer J. , and Metzger J. M. , Duchenne Muscular Dystrophy: Disease Mechanism and Therapeutic Strategies, Frontiers in Physiology. (2023) 14, 1183101, 10.3389/fphys.2023.1183101, 37435300. 37435300 PMC10330733

[26] Song E. , Jaishankar G. B. , Saleh H. , Jithpratuck W. , Sahni R. , and Krishnaswamy G. , Chronic Granulomatous Disease: A Review of the Infectious and Inflammatory Complications, Clinical and Molecular Allergy. (2011) 9, no. 1, 10.1186/1476-7961-9-10. PMC3128843 21624140

[27] Prigent H. , Respiratory Care in Duchenne Muscular Dystrophy, Archives de Pédiatrie. (2025) 32, no. 7, 7S25–7S31, 10.1016/s0929-693x(25)00250-7. 41391907

[28] Allen D. G. , Whitehead N. P. , and Froehner S. C. , Absence of Dystrophin Disrupts Skeletal Muscle Signaling: Roles of Ca2+, Reactive Oxygen Species, and Nitric Oxide in the Development of Muscular Dystrophy, Physiological Reviews. (2016) 96, no. 1, 253–305, 10.1152/physrev.00007.2015. 26676145 PMC4698395

[29] Birnkrant D. J. , Bushby K. , Bann C. M. , Alman B. A. , Apkon S. D. , Blackwell A. , Case L. E. , Cripe L. , Hadjiyannakis S. , Olson A. K. , Sheehan D. W. , Bolen J. , Weber D. R. , Ward L. M. , and DMD Care Considerations Working Group , Diagnosis and Management of Duchenne Muscular Dystrophy, Part 2: Respiratory, Cardiac, Bone Health, and Orthopaedic Management, Lancet Neurology. (2018) 17, no. 4, 347–361, 10.1016/s1474-4422(18)30025-5, 29395990. 29395990 PMC5889091

[30] Daikoku K. , Kondo H. , Kudo M. , and Sugiura A. , A Case of Duchenne Muscular Dystrophy Recovered From Prolonged Ischemic Kidney Injury Which Emerged With a Normal Creatinine Level, CEN Case Reports. (2024) 13, no. 5, 397–402, 10.1007/s13730-024-00858-2. 38436872 PMC11444038

[31] Childs A.-M. , Turner C. , Astin R. , Bianchi S. , Bourke J. , Cunningham V. , Edel L. , Edwards C. , Farrant P. , Heraghty J. , James M. , Massey C. , Messer B. , Michel Sodhi J. , Murphy P. B. , Schiava M. , Thomas A. , Trucco F. , and Guglieri M. , Development of Respiratory Care Guidelines for Duchenne Muscular Dystrophy in the UK: Key Recommendations for Clinical Practice, thorax. (2024) 79, no. 5, 476–485, 10.1136/thorax-2023-220811, 38123347. 38123347 PMC11041593

[32] US Food & Drug Administration , FDA Approves Nonsteroidal Treatment for Duchenne Muscular Dystrophy, 2024, US Food & Drug Administration, http://www.fda.gov/news-events/press-announcements/fda-approves-nonsteroidal-treatment-duchenne-muscular-dystrophy.

[33] US Food & Drug Administration , DUVYZAT (givinostat) Oral Suspension: Prescribing Information. Initial U.S. approval: 2024, 2024, US Food & Drug Administration, http://www.accessdata.fda.gov/drugsatfda_docs/label/2024/217865Orig1s000lbl.pdf.

[34] Guo Z. and Deng S. , Editorial: Current Trends in Muscle Diseases and Their Treatment Strategies, Frontiers in Cell and Developmental Biology. (2025) 13, 1660074, 10.3389/fcell.2025.1660074. 40772232 PMC12325312

